# DNA methylation dynamics during pregnancy

**DOI:** 10.3389/fcell.2023.1185311

**Published:** 2023-05-22

**Authors:** Delphine Fradin, Jorg Tost, Florence Busato, Clémence Mille, Fanny Lachaux, Jean-François Deleuze, Gisèle Apter, Alexandra Benachi

**Affiliations:** ^1^ INSERM U1169, Bicêtre Hospital, Paris Sud University, Le Kremlin-Bicêtre, Paris, France; ^2^ The Laboratory for Epigenetics and Environment, Centre National de Recherche en Genomique Humaine, CEA-Institut de Biologie Francois Jacob, Université Paris-Saclay, Evry, France; ^3^ Centre National de Recherche en Génomique Humaine, Institut de Biologie François Jacob, CEA, Université Paris-Saclay, Evry, France; ^4^ Child and Perinatal Psychiatric Department, Le Havre University Hospital, University Rouen Normandie, Le Havre, France; ^5^ Department of Obstetrics and Gynecology, DMU Santé des Femmes et des Nouveau-nés, Assistance Publique Hôpitaux de Paris, Antoine Beclere Hospital, Université Paris-Saclay, Paris, France

**Keywords:** DNA methylation, pregnancy, changes, bonding, immunity

## Abstract

Pregnancy is a state of multiple physiological adaptations. Since methylation of DNA is an epigenetic mechanism that regulates gene expression and contributes to adaptive phenotypic variations, we investigated methylation changes in maternal blood of a longitudinal cohort of pregnant women from the first trimester of gestation to the third. Interestingly, during pregnancy, we found a gain of methylation in genes involved in morphogenesis, such as ezrin, while we identified a loss of methylation in genes promoting maternal-infant bonding (*AVP* and *PPP1R1B*). Together, our results provide insights into the biological mechanisms underlying physiological adaptations during pregnancy.

## Introduction

DNA methylation, the addition of a CH_3_ group to the carbon-5 position of cytosine bases in the context of CpG dinucleotides, has been associated with multiple adverse health conditions; however, in non-pathological states, DNA methylation is a key determinant of plasticity and adaptation while maintaining normal cellular function and gene expression. DNA methylation is sensitive to numerous extrinsic and intrinsic factors, such as nutrition, stress, sex, or aging (Lam et al., 2012). Some studies also showed that DNA methylation in peripheral blood cells varies over time (Bjornsson et al., 2008; Feinberg et al., 2010; Flanagan et al., 2015). However, little is known about the dynamics of maternal DNA methylation status during pregnancy, even if this period is a well-known state of intense physiological changes. In this sense, DNA methylation would modulate gene expression and thus participate in the gestational adaptation of almost all maternal organs.

This study aimed to fill this gap by investigating DNA methylation changes in maternal blood from early to late pregnancy (up to week 18 of amenorrhea compared with week 35 and later) in 36 Caucasian women.

## Methods

Pregnant women were included in the study in accordance with French bioethics law, with patients being carefully informed and having signed a detailed informed consent. All protocols were approved by the French ethic councils (CPP C0-14-001, CNIL No. C13–61). DNA was extracted from whole blood cells using the Gentra DNA extraction kit (Qiagen). Genomic DNA (1 μg) from each of the 36 pregnant women was bisulfite-converted using the EpiTect^®^ Fast 96 DNA Bisulfite Kit (Qiagen), and the DNA was analyzed using the Illumina Infinium MethylationEPIC (850K) BeadChip at the Centre National de Recherche en Genomique Humaine (CNRGH, CEA, Evry, France). Preprocessing and normalization were performed using the ChAMP pipeline and involved steps of probe filtering, color bias correction, background subtraction, and beta-mixture quantile normalization method (Tian et al., 2017). Array and slide effects were corrected using the ComBat function implemented in the ChAMP package as well (Tian et al., 2017). Cell-type heterogeneity corrected beta matrix and cell-type-specific proportion in each blood sample were obtained using the champ.refbase function again implemented in the ChAMP package. Finally, we used this same package to identify differentially methylated CpG (DMP) and differentially methylated regions (DMRs) using the BumpHunter algorithm (Tian et al., 2017). Functional enrichment analysis was conducted on the GSEA website (http://www.gsea-msigdb.org/).

## Results

DNA methylation variation was explored in 36 pregnant women. The age of the women ranged from 24.1 to 38.1 years (median 30.31), with a first-trimester BMI of 23.45 ± 3.42 kg/m^2^ and a weight gain of 8.91 ± 0.2 kg during pregnancy (Supplemental Figure 1). We analyzed DNA methylation on the Infinium Human MethylationEPIC Beadchip (Illumina) that covers 846,604 CpG loci at single-nucleotide resolution.

As cellular heterogeneity strongly influences methylation profiles and can drive some of the methylation differences detectable across individual blood samples, we first estimated the cell-type compositions in each sample. We found that cellular composition varies across individuals and across the first and third trimesters, so we then used the corrected beta matrix to conduct our analyses and avoid those differences in methylation resulting from differences in cellular heterogeneity.

**FIGURE 1:**
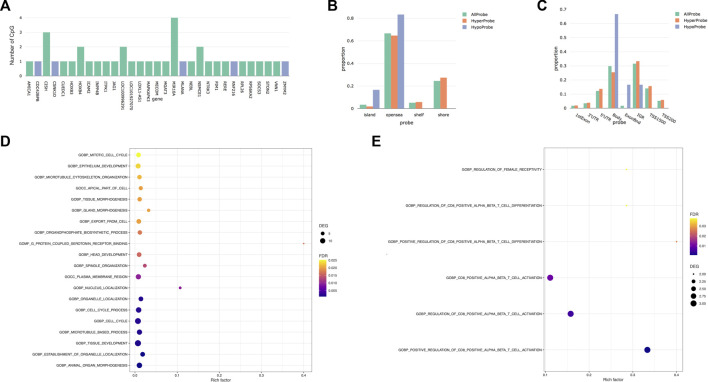
Differentially methylated CpGs between first and third trimesters. **(A)** Number of differentially methylated CpGs by genes. Blue represents the hypomethylated genes, while green represents the hypermethylated genes. **(B)** Distribution of differentially methylated CpGs *versus* all CpG sites on the EPIC array in relation to the CpG island annotation. **(C)** Distribution of differentially methylated CpGs *versus* all CpG sites on the EPIC array in relation to the nearest gene regions. ExonBnd, exon binding region; IGR, intergenic region; TSS200, 200 base pairs (bps) around the transcriptional start site; TSS1500, 1500 bps around the transcriptional start site. **(D)** Bubble diagram of Gene Ontology (GO) analysis of DMRs that gained methylation across pregnancy. **(E)** Bubble diagram of Gene Ontology (GO) analysis of DMRs that lose methylation during pregnancy. The *Y*-axis label represents the pathway, and the *X*-axis label represents the rich factor (rich factor = amount of differentially expressed genes enriched in the pathway/amount of all genes in the background gene set). Size and color of the bubble represent the amount of differentially expressed genes enriched in the pathway and enrichment significance, respectively. DEGs, differentially expressed genes; FDR, false discovery rate.

After correction for cellular heterogeneity and multiple testing, methylation levels changed at 57 CpGs between the first and third trimesters of pregnancy (Table 1; Supplementary Table S1). These CpGs are annotated to 31 genes, and five showed an increasing methylation level during pregnancy, whereas all others decreased. Among them, *MIR10A* showed methylation changes at four distinct CpGs (Figure 1A). No hypermethylated CpGs were located in shore or shelf regions, whereas we observed an enrichment of these CpGs over the CpG islands (CGIs) or the opensea regions (Figure 1B). The genomic distribution of the 57 CpGs in comparison to all the probes located on the 850K BeadChip array with respect to the gene structure showed an enrichment of hypermethylated CpGs inside the gene body or exon binding regions (Figure 1C). We found no correlation between these DNA methylation changes and age or BMI in our cohort (Supplementary Figure S2).

**TABLE 1:** Top 10 of the most significant differentially methylated CpGs and the most significant differentially methylated regions (DMRs) during pregnancy.

	Chromosome	Position	Gene	Feature	CGI	Adjusted *p*-value
cg13652985	17	46658257	*MIR10A*	TSS1500	Shore	3.11e-05
cg00052692	12	122442863		IGR	Opensea	0.00029
cg06123699	3	188817035		IGR	Opensea	0.00029
cg08996521	3	50649994	*CISH*	TSS1500	Shore	0.00029
cg15885703	11	118094830	*AMICA1*	5′UTR	Opensea	0.00062
cg05844798	20	3062344		IGR	Shore	0.00069
cg09430344	16	27237355	*NSMCE1*	Body	Opensea	0.00088
cg03067296	17	76274577	*LOC100996291*	TSS200	Opensea	0.00179
cg20701457	17	62084342	*ICAM2*	5′UTR	Opensea	0.00179
cg11047325	17	76354934	*SOCS3*	Body	Island	0.00378
DMR1	6	33048310–33048919	*HLA-DPA1*			3.655e-05
DMR2	17	79004850–79005662	*BAIAP2-AS1*			5.649e-05
DMR3	20	3065343–3065698	*AVP*			5.6491e-05
DMR4	6	30458998–30459867	*HLA-E*			7.310e-05
DMR5	21	36259067–36259797	*RUNX1*			7.311e-05
DMR6	6	30130819–30131283	*TRIM15*			7.975e-05
DMR7	17	8702486–8702896	*MFSD6L*			0.00014
DMR8	5	54281198–54281733	*ESM1*			0.00011
DMR9	1	870791–871546	*SAMD11*			0.00012
DMR10	4	81118188–81118794	*PRDM8*			0.00015

Next, we analyzed our DNA methylation data to identify DMRs, since it has been shown that the identification of regional differences across several CpGs provides more robust findings and is more likely to be replicated than individual CpG differences. We identified 157 DMRs between the first and third trimesters of pregnancy (Table 1; Supplementary Table S2); 98 DMRs showed a gain of methylation, while 59 DMRs showed a decrease. Gene Ontology (GO) analysis identified enrichment in several pathways involved in tissue development, morphogenesis, and cell cycle in the 98 DMRs that gained methylation during pregnancy (Figure 1D), whereas those that lost methylation were involved in immune cell functions (Figure 1E).

## Discussion

Our study supports evidence that DNA methylation changes in maternal blood cells during pregnancy, which may be important for maternal adaptation to gestation and labor. Indeed, maternal systems progressively adapt during human pregnancy to accommodate the increasing demands of fetal growth and development and prepare for parturition. These physiological adaptations seem to correlate with DNA methylation changes as a master regulator of gene expression.

We identified 39 individual CpGs and 157 regions showing DNA methylation changes across pregnancy after correction for multiple testing. Biological pathway analysis revealed that CpGs that gained methylation included genes involved in organ morphogenesis, epithelium development, and cell cycle, among others. These genes are needed during the formation of the placenta or the early stages of pregnancy. Among them, ezrin (*EZR*) is an interesting candidate since it is a cytoskeletal linker protein that facilitates uterine receptivity and embryo–endometrium physical attachment (Lecce et al., 2011). Its expression is consequently necessary at the beginning of the pregnancy and seems to be regulated by DNA methylation (Turco et al., 2018).

Concerning CpGs that lose DNA methylation during pregnancy, they belong mainly to genes involved in immune functions. It is indeed well described that the maintenance of pregnancy relies on finely tuned immune adaptations since the maternal immune system should tolerate the fetus while preserving its abilities against microbial or viral attacks. We interestingly identified a loss of DNA methylation in *HLA-E* (human leukocyte antigen E). This gene was initially recognized as *HLA-6.2* and is a ligand of KIR (killer cell immunoglobulin-like receptor) of NK (natural killer) cells, which leads to downregulation of immune response and helps in the maintenance of pregnancy. In this context, Tripathi et al. (2006) found that expression, affinity, and stability of some *HLA-E* alleles were associated with the success of pregnancy.

Finally, we identified two genes (*AVP*, arginine vasopressin and *PPP1R1B*, protein phosphatase 1 regulatory (inhibitor) subunit 1B) which lose DNA methylation during pregnancy and are linked to “regulation of female receptivity.” These genes are indeed fascinating since they are associated with maternal-infant bonding. Indeed, AVP is a neuropeptide with crucial regulatory functions in a wide spectrum of socio-emotional and cognitive processes in humans and animals. Research on prairie voles has highlighted the role of AVP in attachment and has also shown its epigenetic regulation (Sadino and Donaldson, 2018). PPP1R1B, also called DARPP-32*,* plays an essential role in mediating dopamine effects. Moreover, maternal neglect was linked to dysregulation of dopamine transmission, associated itself with a modified DARPP-32 phosphorylation pattern (Scheggi et al., 2018). The demethylation of these two genes during pregnancy indicates that they are probably highly expressed before delivery to enhance the capacity for nursing and maternal care of the newborn. It is important to note, however, that our observations were made in blood, so we can only speculate that this is also true in the brain, where these two genes are expressed.

Several studies have previously identified age-associated changes in DNA methylation in blood cells (Hannum et al., 2013; Horvath, 2013; Johansson et al., 2013; Florath et al., 2014), which led to the development of epigenetic clocks (Horvath and Raj, 2018). The small age range used in our study likely explains the lack of correlation observed in our cohort between age and DNA methylation since 19 of our 57 identified CpGs have previously been associated with chronological age in a large cohort of individuals aged 14–94 years (Johansson et al., 2013). Similarly, many studies have reported BMI-related differential methylation CpG sites, whereas no correlation was found in our cohort (Dick et al., 2014; Aslibekyan et al., 2015; Demerath et al., 2015; Huang et al., 2015; Fradin et al., 2017; Mendelson et al., 2017). Only cg17501210, associated with methylation changes during pregnancy in our study, was linked to BMI in three independent cohorts (ARIC: Atherosclerosis Risk in Communities, GOLDN: Genetics of Lipid Lowering Drugs and Diet Network, and PIVUS: Prospective Investigation of the Vasculature in Uppsala Seniors) (Mendelson et al., 2017).

Consistently, using the same EPIC BeadChip technology, Gruzieva et al. (2019) also observed DNA methylation changes at several CpGs within *MIR10A*, *LOC100996291*, and *AVP* in 21 pregnant women. Only one other study investigated DNA methylation in maternal blood during pregnancy and, by contrast, showed that DNA methylation becomes stable over time. However, they analyzed approximately 200 000 CpGs (Chen et al., 2017). We cannot exclude the possibility that our identified CpGs were not present in their less covering array.

Our study has several limitations. The first limitation is the small sample size, which can produce false-positive results. However, the fact that some identified CpGs were previously described in the only other study investigating DNA methylation changes in maternal blood during pregnancy reinforces our results. The second limitation is that we conducted our analysis by considering the blood cell heterogeneity but not fetal cells that may be present in maternal circulation, although in very low proportions.

Altogether, our results showed that DNA methylation may play a role not only in maternal adaptations during pregnancy and preparation for delivery but also in adaptation to care for the newborn.

## Data Availability

The datasets presented in this study can be found in online repositories. The names of the repository/repositories and accession number(s) can be found at: https://www.ncbi.nlm.nih.gov/geo/, GSE224339.
