# Supplementary figures and images for: DNA methylation dynamics during pregnancy
Source: Front Cell Dev Biol. 2023 May 22;11:1185311. doi: 10.3389/fcell.2023.1185311 (PMC10242503; doi:10.3389/fcell.2023.1185311)

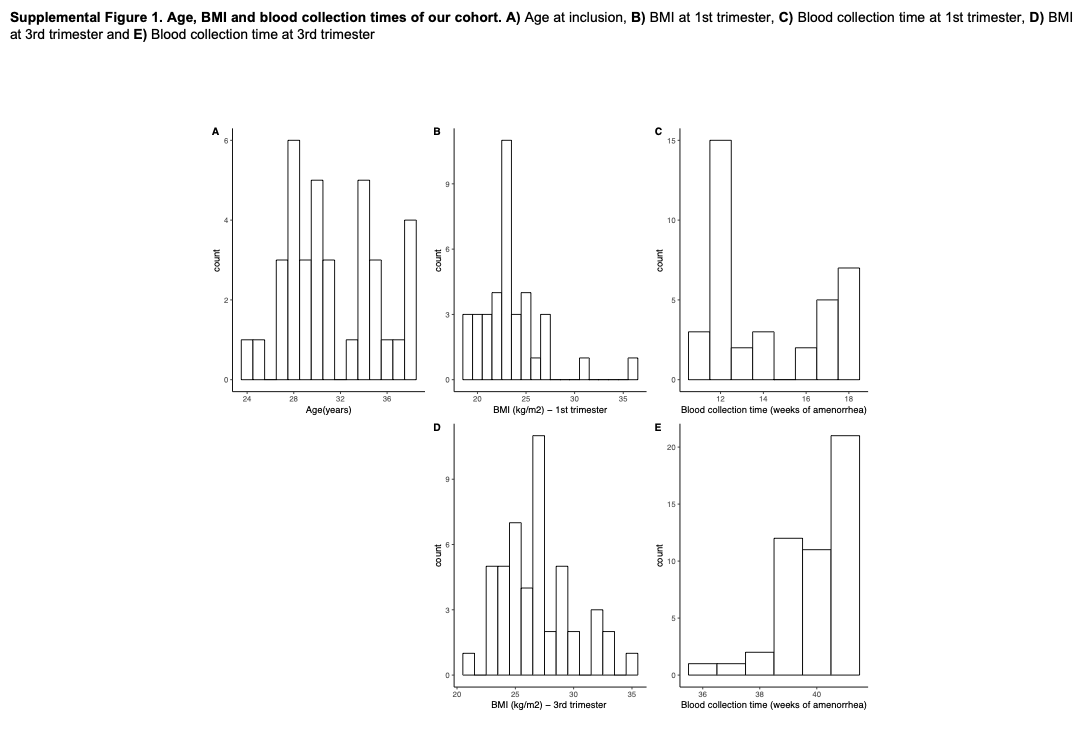

Supplement: Supplementary file 1 [file Image1.TIFF]
